# Multi-parametric Computer Tomographic Imaging of Left Ventricular Free Wall Rupture Post-Myocardial Infarction

**Published:** 2022

**Authors:** Mohamed Abdelgader, Monika Arzanauskaite, Jurate Noreikaite, Aleem Khand

**Affiliations:** 1 Department of Cardiology, Liverpool University Hospital NHS trust, Lower Lane Liverpool University Hospitals NHS Trust, Lower Lane, Liverpool, UK; 2 Radiology and Imaging Department, Liverpool Heart and Chest Hospital, Liverpool, Thomas Drive, Liverpool, UK

## INTRODUCTION

Left ventricular free wall rupture (LVFWR) is an uncommon yet catastrophic complication of myocardial infarction (MI). The protean manifestation of LVFWR and the challenging imaging in patients in extremis and the critical time-window for surgical intervention pose considerable challenges. Here in, we report a case where multiparametric CT was utilised to elucidate the diagnosis, define coronary anatomy and aid strategy in emergency cardiothoracic surgery for LVFWR.

## CASE DESCRIPTION

**Figure 1:**
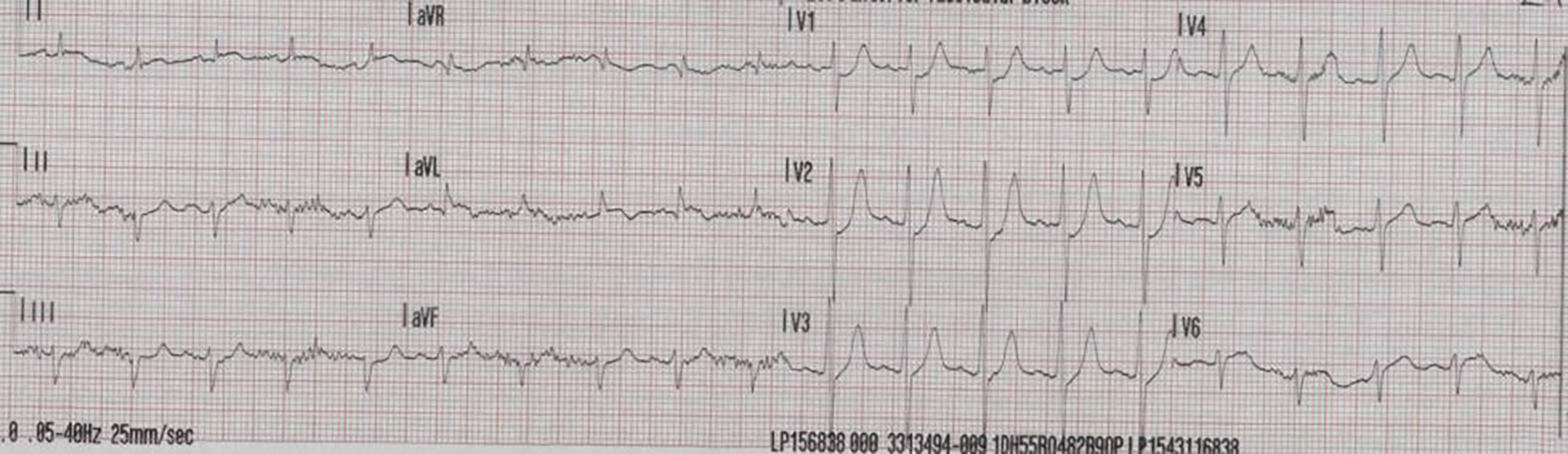
12 Lead ECG showing minimal ST elevation in the lateral leads.

**Figure (2A - 2I):**
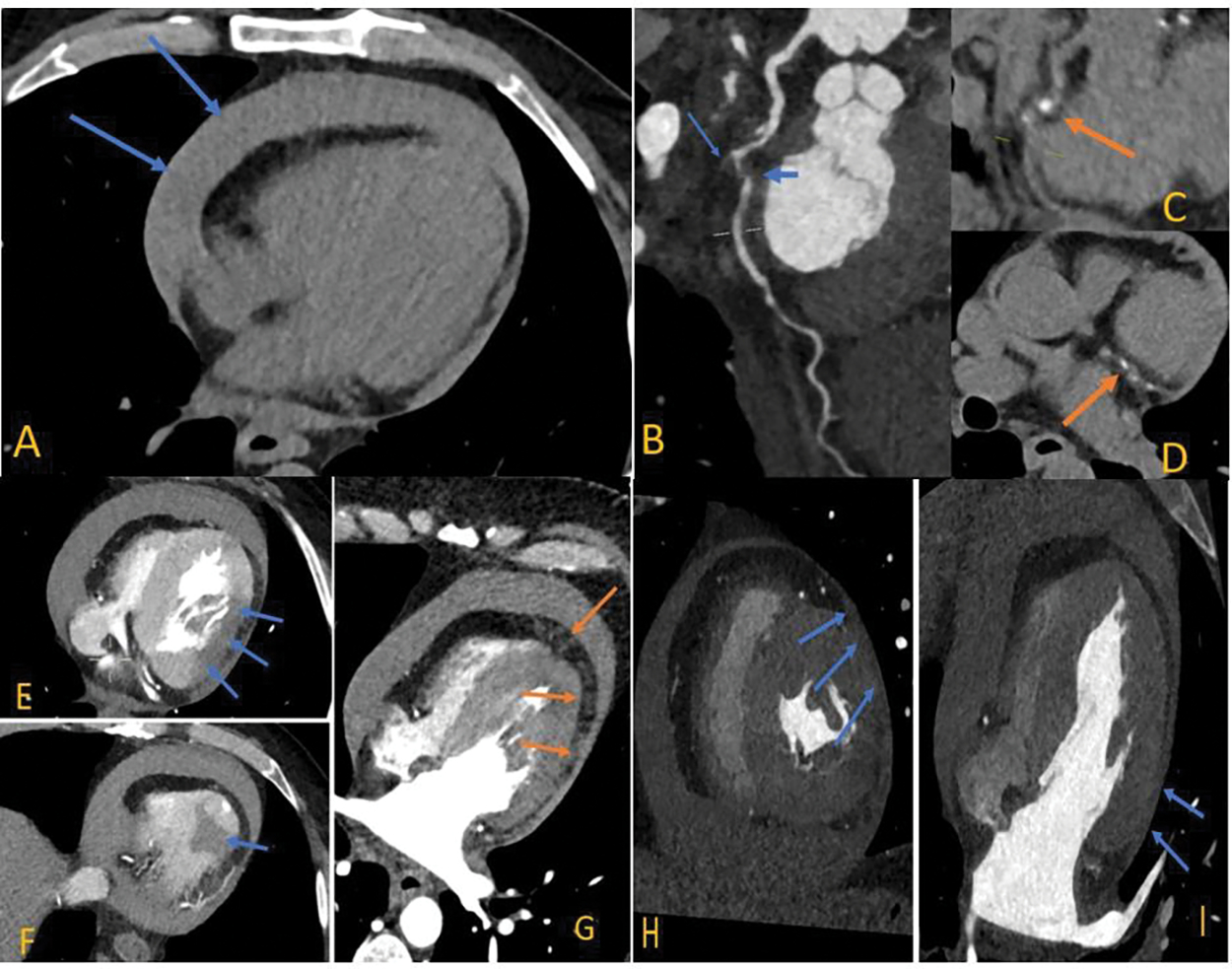
Multi-parametric CT imaging of left ventricular free wall rupture.

A 59-years-old male, with controlled hypertension collapsed at home and was brought into hospital in extremis with an unrecordable blood pressure, however, remained conscious and denied chest pain. Fluid resuscitation and intravenous boluses of adrenaline increased systolic blood pressure to 80 mmhg. His 12-lead electrocardiogram (ECG) revealed tachycardia with minimal ST elevation in the lateral leads (Figure 1). Portable transthoracic echocardiography showed cardiac tamponade with clots in the pericardial fluid but there was no clear primary pathology such as myocardial rupture, left ventricular (pseudo) aneurysm or aortic dissection identified. Emergency pericardiocentesis was not undertaken due to concerns relating to a possible aortic dissection or myocardial rupture. An emergency CT aorta was undertaken to help define the primary pathology (Figure 2A – Figure 2I).

## DISCUSSION

The unenhanced phase of the CT showed a medium-sized to large pericardial effusion of high attenuation, in keeping with a haemopericardium (Figure 2A). Multiplanar reconstruction of the arterial phase images revealed an occluded first obtuse marginal branch (OM1) of the left circumflex artery (LCx) (Figure 1, B long arrow) and a significant stenosis in the mid LCx (Figure 2, B short arrow). At this level, there was also epicardial fat stranding along the atheromatous vessels with mixed plaques (Figure 2B; the calcification was better appreciated on the unenhanced phase, C and D).

The arterial phase of the scan fully excluded an acute aortic syndrome, but showed diminished enhancement (Figure 2E & Figure 2F; arrowed) in the lateral and inferior wall of the left ventricle (LV), suggestive of acute myocardial infarction1, 2. In addition, a multiplanar reconstruction (Figure 2G) showed small pockets of fluid in the epicardial fat along the basal to apical LV wall, and following the course of the LCx artery. While the exact site of LV rupture was not evidenced by contrast extravasation, the fluid pockets were thought to represent blood leaking into the epicardial tissue.

Finally, on CT, there was adhesion of the pericardium to the LV lateral wall at the territory of the OM1 (note the lack of epicardial adipose tissue layer next to the LV lateral wall) with no thinning of the myocardium indicating a type III LVFW3 (Figure 2H & Figure 2I). The lateral wall appeared non-viable and therefore no attempt was made to perform coronary artery bypass to the circumflex and obtuse marginal branch.

**Figure (3A and 3B):**
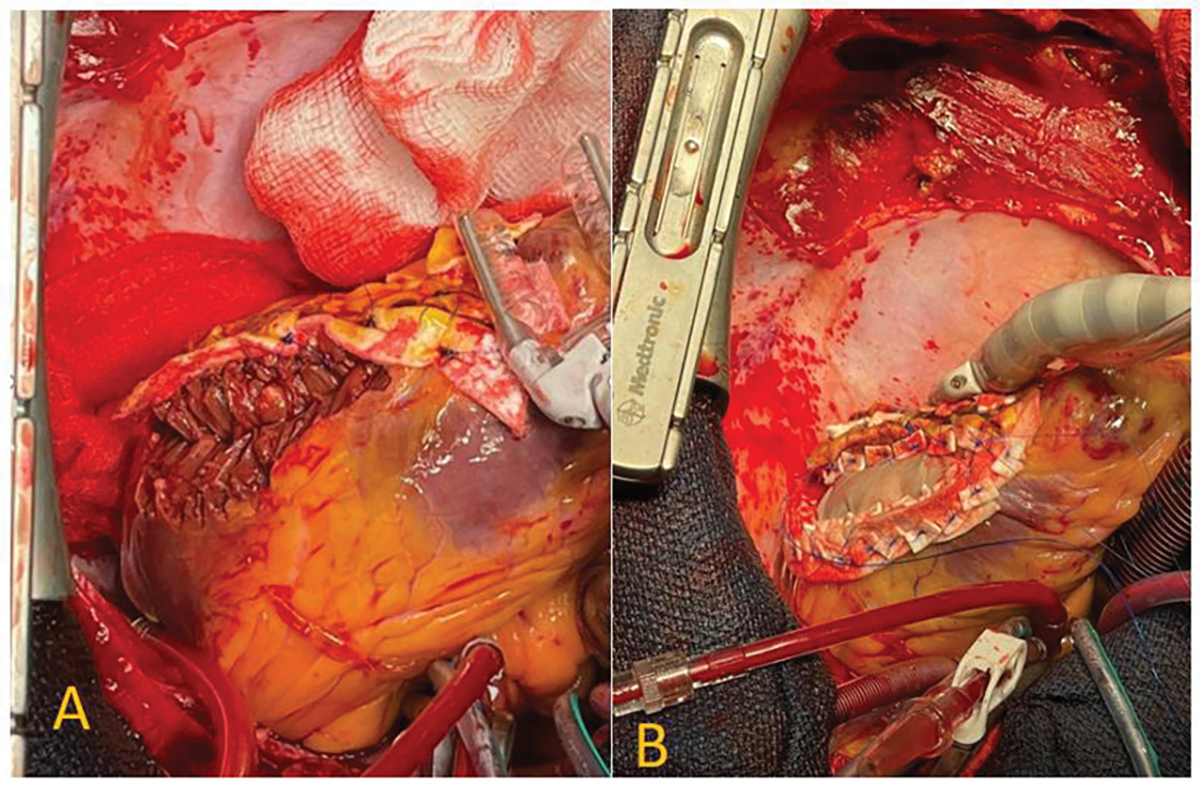
Intra-operative images of the LVFWR.

Despite haemodynamic support, systolic BP remained at 70 mmhg. Emergency cardiac surgery was undertaken. Intraoperatively, the pericardium was under tense pressure with five holes in the lateral wall of the myocardium and about 200mls of blood in the pericardial space. The lateral LV wall was successfully repaired using two bovine pericardial patches (Figure 3A & Figure 3B).

## CONCLUSION

The aetiology of pericardial tamponade was not apparent on baseline echocardiography, admittedly in a critically ill patient. Urgent CT imaging revealed the primary pathology, including the culprit coronary lesion and the corresponding myocardial infarction [1,2] and aided in planning the emergency cardiothoracic surgery. The CT scan also indicated a type III LVFW rupture without thinning of the myocardium and with an evident pericardial adhesion [3]. The lateral wall appeared non-viable and therefore no attempt was made to perform coronary artery bypass but simply to repair the ruptured myocardium.

This case demonstrates the use of urgent CT in suspected cases of LVFWR, where it can elucidate in diagnosis and categorisation of LVFWR, identify the culprit lesion and aid strategy in emergency cardiothoracic surgery as well as revascularisation.
